# Uropathogenic *Escherichia coli* in a Diabetic Dog with Recurrent UTIs: Genomic Insights and the Impact of Glucose and Antibiotics on Biofilm Formation

**DOI:** 10.3390/microorganisms13081946

**Published:** 2025-08-20

**Authors:** Inês C. Rodrigues, Marisa Ribeiro-Almeida, Joana Campos, Leonor Silveira, Liliana Leite-Martins, Jorge Ribeiro, Paula Martins da Costa, Joana C. Prata, Ângela Pista, Paulo Martins da Costa

**Affiliations:** 1 School of Medicine and Biomedical Sciences, University of Porto (ICBAS-UP), Rua de Jorge Viterbo Ferreira, 228, 4050-313 Porto, Portugal; icrodrigues@icbas.up.pt (I.C.R.); mra.microvet@gmail.com (M.R.-A.); jacampos@icbas.up.pt (J.C.); lmartins@icbas.up.pt (L.L.-M.); jribeiro@icbas.up.pt (J.R.); 2 Interdisciplinary Centre of Marine and Environmental Research (CIIMAR), Terminal de Cruzeiros do Porto, de Leixões, Av. General Norton de Matos s/n, 4450-208 Matosinhos, Portugal; 3 UCIBIO—Applied Molecular Biosciences Unit, Laboratory of Microbiology, Department of Biological Sciences, Faculty of Pharmacy, University of Porto, 4050-013 Porto, Portugal; 4 National Reference Laboratory for Gastrointestinal Infections, Department of Infectious Diseases, National Institute of Health Doutor Ricardo Jorge, Av. Padre Cruz, 1649-016 Lisbon, Portugal; leonor.silveira@insa.min-saude.pt (L.S.); angela.pista@insa.min-saude.pt (Â.P.); 5 Microbiology Department, Centro Hospitalar Universitário do Porto, Largo do Prof. Abel Salazar, 4099-001 Porto, Portugal; pcmcosta@live.com.pt; 6 i4HB—Institute for Health and Bioeconomy, Institute of Health Sciences—CESPU, 4585-116 Gandra, Portugal; joanacorreiaprata@gmail.com; 7 UCIBIO—Applied Molecular Biosciences Unit, Translational Toxicology Research Laboratory, Institute of Health Sciences (1H-TOXRUN, IUCS-CESPU), 4585-116 Gandra, Portugal

**Keywords:** Uropathogenic *Escherichia coli*, UTI, antibiotic pressure, biofilm

## Abstract

Recurrent urinary tract infections (UTIs) pose a significant clinical challenge in both human and veterinary medicine, due to antibiotic-resistant and biofilm-forming bacteria. We hypothesized that high glucose levels in diabetic animals enhance biofilm formation and reduce antibiotic efficacy, promoting infection persistence. This study analyzed *Escherichia coli* from a diabetic female Labrador Retriever with recurrent UTIs over 18 months, focusing on antimicrobial resistance, biofilm-forming capacity, and genomic characterization. Most isolates (9/11) were resistant to ampicillin and fluoroquinolones. Whole genome sequencing of six selected isolates revealed that they belonged to the multidrug-resistant ST1193 lineage, a globally emerging clone associated with persistent infections. Phylogenetic analysis revealed clonal continuity across six UTI episodes, with two distinct clones identified: one during a coinfection in the second episode and another in the last episode. High-glucose conditions significantly enhanced biofilm production and dramatically reduced antibiotic susceptibility, as evidenced by a marked increase in minimum biofilm inhibitory concentrations (MBICs), which were at least 256-fold higher than the corresponding minimum inhibitory concentration (MIC). Sulfamethoxazole–trimethoprim demonstrated the strongest antibiofilm activity, though this was attenuated in glucose-supplemented environments. This research highlights the clinical relevance of glucosuria in diabetic patients and emphasizes the need for therapeutic strategies targeting biofilm-mediated antibiotic tolerance to improve the management of recurrent UTIs.

## 1. Introduction

Uropathogenic *Escherichia coli* (UPEC) is a major pathogen causing urinary tract infections (UTIs) in both humans and animals. Among the human population, 80% of UTIs are caused by UPEC strains [1]. UPEC is categorized as a subpathothype of extra-intestinal pathogenic *Escherichia coli* (ExPEC), demonstrating the ability to cause disease associated with the expression of several virulence factors, such as lipopolysaccharide (LPS), polysaccharide capsule, flagella, outer-membrane vesicles, pili, curli, non-pilus adhesins, outer membrane proteins (OMPs), toxins, and iron-acquisition systems [2,3]. Furthermore, UPEC is mainly classified into phylogenetic group B2 (and a few in group D), which is associated with antimicrobial susceptible isolates with higher virulence-associated gene prevalence [4]. Additionally, isolates belonging to Sequence Type (ST) 14, ST69, ST73, ST95, and ST131 are the predominant STs responsible for most extra-intestinal infections in humans and have also been identified in companion animals [5,6,7].

Biofilm production by UPEC plays a crucial role in the establishment, persistence, and recurrence of UTIs, providing protection against adverse conditions (e.g., pH variations, oxidative stress, nutrient limitation), antimicrobial agents, and the host’s immune defenses [8,9]. Diabetes mellitus is a recognized risk factor for the increased prevalence of bacterial UTIs in dogs and is frequently associated with recurrent or persistent infections [10]. This process may be attributed to common complications of diabetes, including glucosuria, nephropathy, and immune dysregulation, which impair both the host’s capacity to combat infections and the effectiveness of treatment, thereby promoting infection persistence and tolerance and increasing the risk of antimicrobial resistance (AMR) [11].

UTIs are among the most important indications for antimicrobial use in veterinary medicine [12,13,14,15,16]. Unfortunately, these drugs are still frequently prescribed inappropriately for reasons unaligned with clinical practice guidelines [17]. Over time, this misuse and overuse contributed to the emergence and dissemination of AMR, raising serious concerns regarding therapeutic efficacy and broader implications for public health [18,19]. This issue is particularly relevant in the context of companion animals, where close and frequent contact with humans, combined with the use of similar antimicrobial classes in both human and veterinary medicine, increases the potential for cross-species transmission of resistant bacteria [20,21]. A recent report indicated that broad-spectrum and critically important antimicrobials for human medicine accounted for 83% and 71% of total antimicrobial treatments administered to dogs and cats, respectively [22,23].

In response to the growing threat of AMR, antimicrobial stewardship has gained increasing importance in veterinary medicine, promoting the prudent and evidence-based use of antimicrobials [19,24]. International recommendations, such as World Health Organization (WHO) Global Action Plan and European Medicines Agency (EMA) antimicrobial categorization list have influenced the development of Regulation (EU) 2022/1255, which restricts certain antibiotic classes of antimicrobials exclusively for human use [25,26,27]. This regulatory framework represents a major step toward restricting empirical prescribing and aligning veterinary practices with One Health priorities. Nevertheless, therapeutic options for UTIs in companion animals remain limited, as many of the most effective agents, such as fluoroquinolones and cephalosporins, are classified by the WHO as critically important for human health [28]. This reality underscores the need for strict adherence to clinical guidelines and the responsible use of first-line antimicrobials to limit the further emergence and spread of AMR [12].

Recurrent UTIs in diabetic dogs are therapeutically challenging, particularly in the context of increasing AMR and limited treatment options. Yet, there is limited data on the genomic and phenotypic features of UPEC in diabetic dogs with recurrent UTIs, especially regarding their biofilm-forming ability. Glucosuria in diabetic dogs may create a glucose-rich urinary environment that enhances biofilm-mediated antibiotic tolerance, contributing to infection persistence. While the roles of hyperglycemia, glucosuria, and biofilms in UTIs are known, their combined impact on antimicrobial tolerance in veterinary medicine is poorly understood. Therefore, the aim of this study was to phenotypically and genotypically characterize UPEC isolates recovered from a diabetic dog with recurrent UTIs and to explore the impact of glucose on biofilm production in the presence of antibiotics administered during the dog’s treatment. The findings intend to provide critical insights to support clinical decisions, optimize antimicrobial strategies, and address challenges associated with persistent infections in similar clinical contexts.

## 2. Materials and Methods

### 2.1. Clinical Case Selection

From 1 January to 31 December 2023, cases admitted to the Veterinary Hospital (UPVet) of the Institute of Biomedical Sciences Abel Salazar, University of Porto (ICBAS/UP), were reviewed (*n* = 9247). In the present study, eligibility criteria for case inclusion comprised the following: (i) admission to UPVet for UTI diagnosis; (ii) follow-up at UPVet; (iii) submission of more than one consecutive sample to the microbiology laboratory of ICBAS-UP (microLAB) during the same infectious episode; (iv) availability of complete antibiotic therapy records; and (v) confirmation of recurrent UTI. A single case was selected based on these criteria. All UPVet clients provided informed consent for the use of patient data for scientific research and educational purposes. Dataset was securely stored and anonymized in accordance with Data Protection laws (Regulation (EU) 2016/679).

### 2.2. Microbiological Analysis of Clinical Samples

#### 2.2.1. Urine Collection, Processing, and Bacterial Identification

Urine samples, each with a minimum volume of 2 mL, were collected by cystocentesis and promptly transported within 2 h (h) to the laboratory of Microbiology of ICBAS/UP. Aseptically, 100 µL of each sample was inoculated into Tryptic Soy Agar (TSA, Biokar, Allone, France) supplemented with 5% (*v*/*v*) defibrinated horse blood (Oxoid, Basingstoke, UK). Simultaneously, 100 µL was plated onto MacConkey Agar (MAC, Biokar), while 100 µL was added to brain heart infusion broth (BHI, Biokar). In addition, to estimate the bacterial load in urine samples, serial ten-fold dilutions were prepared and plated on both MAC and blood agar. Following inoculation, plates were aerobically incubated at 37 °C for 24 h, whereas BHI was incubated for at least 48 h. From each culture plate, at least three colonies were selected for further analysis. The presumptive identification of isolates was based on colony morphology, Gram staining, and the oxidase test (Liofilchem, Roseto degli Abruzzi, Italy). Further phenotypic characterization included glucose and lactose fermentation using Triple Sugar Iron (TSI, Biokar) agar and assessment of motility, urease activity, and indole production using Motility Indole Urea (MIU, Biokar) medium. Bacterial species identification was confirmed using the RapID™ ONE System (Remel Inc., Thermo Fisher Scientific, Waltham, MA, USA), following the manufacturer’s instructions.

If no growth was observed on the plates but turbidity was detected in BHI, fluorescence in situ hybridization (FISH) was conducted following the method previously described [29]. Initially, a volume of 50 µL of urine sample was fixed with 4% paraformaldehyde and dehydrated using successive alcohol solutions of increasing concentration. Subsequently, the fixed cells underwent hybridization in a 100 mL buffer solution containing 0.9 M sodium chloride, 0.1% sodium dodecyl sulfate, 20 mM Tris-HCl (pH 7.2), and 1.5 ng/µL of Eco440 probe (TCCCTTCCTCCCCGCTG), specific for *E. coli* detection, at 46 °C for 3 h [29]. Previous studies have demonstrated the specificity and validation of the Eco440 probe [29,30]. Following hybridization, the slides were washed for 30 min at 46 °C and dried. Then, they were mounted using Vectashield^®^ Mounting Medium (Vector Laboratories, Newark, CA, USA) and observed under a Nikon Eclipse E400 microscope (Nikon Instruments, Amsterdam, The Netherlands) at 1000× magnification with an oil immersion objective.

#### 2.2.2. Disk Diffusion Method

The susceptibility profile of each *Escherichia coli* (*E. coli*) isolate was determined following European Committee on Antimicrobial Susceptibility Testing (EUCAST) guidelines [31,32], with additional considerations from bacteria isolated from the animals’ section for marbofloxacin and enrofloxacin antibiotics [33]. Briefly, bacterial inoculum equivalent to 0.5 McFarland turbidity was inoculated in Mueller–Hinton Agar (MHA, Biokar, Allone, France). Subsequently, antibiotic disks were placed on the agar surface, encompassing amoxicillin–clavulanate (AMC, 20/10 µg), amikacin (AMK, 30 µg), ampicillin (AMP, 10 µg), aztreonam (ATM, 30 µg), cefazoline (CFZ, 30 µg), cefoxitin (FOX, 30 µg), cefotaxime (CTX, 30 µg), ceftazidime (CAZ, 30 µg), chloramphenicol (CHL, 30 µg), ciprofloxacin (CIP, 5 µg), doxycycline (DOX, 30 µg), enrofloxacin (ENR, 5 µg) gentamycin (GEN, 10 µg), imipenem (IPM, 10 µg), marbofloxacin (MAR, 5 µg), nitrofurantoin (NIT, 300 µg), trimethoprim–sulfamethoxazole (SXT, 1.25/23.75 µg), tetracycline (TET, 30 µg), and tobramycin (TOB, 10 µg). All antibiotic disks, with the exception of marbofloxacin obtained from Liofilchem, were sourced from Oxoid (Basingstoke, UK). Following incubation for 18–20 h at 37 °C, the inhibition zones’ diameters were measured in millimeters. *E. coli* ATCC 25922 was used as a reference strain.

#### 2.2.3. Whole Genome Sequencing (WGS) Characterization and Bioinformatics Analysis of Six Isolates

Among the eleven isolates obtained from the dog, six were selected for WGS characterization based on their clinical and microbiological relevance. This subset included early isolates defining the onset and recurrence of the infection, as well as the final isolate recovered during the study period, which exhibited a distinct antimicrobial susceptibility profile. The remaining isolates shared identical phenotypic and resistance profiles and therefore were not sequenced.

High-quality DNA samples, extracted using ISOLATE II Genomic DNA Kit (Bioline, London, UK) and quantified by dsDNA HS Assay Kit (Thermo Fisher Scientific, Waltham, MA, USA), were subjected to the NexteraXT library preparation protocol (2 × 250 bp or 2 × 500 bp; Illumina, San Diego, CA, USA) and sequenced on a MiSeq or a NextSeq instrument (Illumina, San Diego, CA, USA), according to the manufacturer’s instructions.

The raw reads were submitted to the QAssembly pipeline (v3.61) of EnteroBase (https://enterobase.warwick.ac.uk/; accessed on 20–27 May 2023 and 26 May 2024) for quality control, trimming, and generating assemblies of high quality. Online bioinformatic tools from EnteroBase were used to determine in silico *E. coli* serotyping, phylogroups, and ST. The raw reads were also submitted to the Centre for Genomic and Epidemiology (CGE, http://www.genomicepidemiology.org, accessed on 5 May 2025) to assess in silico *E. coli* virulence genes (VirulenceFinder 2.0), resistance genes and point mutations (ResFinder 4.7.2) (double-checked by the submission of assemblies), serotypes (SeroTypeFinder 2.0), ST (MLST 2.0), and plasmid replicons (PlasmidFinder 2.1) [34,35]. The presence of two or more UPEC virulence genes were used for the classification of this pathotype [36,37,38].

Comparative phylogenetic analysis was performed using the Enterobase cgMLST V1 + HierCC V1 for *E. coli* (2513 loci) [39]. Phylogenetic trees were generated with GrapeTree software (v1.5.0) employing the minimal spanning tree (MST) algorithm, which visualizes allelic differences among cgMLST profiles to infer phylogenetic relationships [40].

Sequence reads were deposited in the European Nucleotide Archive (ENA) under the bioprojects PRJEB54735.

### 2.3. In Vitro Assessment of Glucose and Antibiotic Effects on Selected Bacterial Isolates

#### 2.3.1. Bacterial Selection

Four isolates were selected for in vitro assays to evaluate the influence of glucose and antibiotics, based on WGS results and their involvement in co-infections. Additionally, *E. coli* ATCC 25922 was used as a reference strain to provide a comparative baseline for interpreting the phenotypic responses of clinical isolates. The use of this strain as a control was preferred for the following reasons: (i) a well-characterized reference strain aligns with standard microbiological practices and facilitates comparability across studies; (ii) under routine veterinary practice, urine samples are rarely collected from clinically healthy animals; and (iii) urine from healthy animals without clinical signs of urinary tract infection generally does not contain *E. coli*.

This selection enabled a focused investigation of the effects of glucose and antibiotics on minimal inhibitory concentrations (MIC), minimum biofilm inhibitory concentration (MBIC) and biofilm production, generating data to support clinical decisions and optimize therapeutic strategies for managing persistent infections.

#### 2.3.2. Antibiotics Selection

Although ampicillin (AMP) was initially considered, it was not used consistently in the therapeutic regimen due to the resistance profiles of the isolates and was ultimately replaced by other antibiotics. Consequently, AMP was excluded from the in vitro assays. Therefore, experimental analysis focused on the antibiotics that were administered as part of complete therapeutic courses: amoxicillin–clavulanate (AMC), cefalexin (CEF), nitrofurantoin (NIT) and trimethoprim–sulfamethoxazole (SXT).

#### 2.3.3. MIC Assays

MICs were recorded for AMC, CEF, NIT, and SXT, reflecting the antibiotics administered during the dog’s treatment. The concentration ranges were selected to accurately determine susceptibility or resistance according to EUCAST clinical breakpoints [32]. The tested concentration ranged as follows: 64/32–1/0.5 µg/mL for AMC, 64–2 µg/mL for CEF, 64–0.25 µg/mL for NIT, and 64/1216–0.015/0.30 µg/mL for SXT. MICs were assessed using the broth microdilution method and interpreted based on EUCAST guidelines and breakpoints [32,41]. Fresh colonies were suspended in cation-adjusted Mueller–Hinton broth (CAMHB, Sigma-Aldrich, St. Louis, MO, USA) to achieve a final inoculum size closely approximated 5 × 10^5^ CFU/mL. Each well of 96-well U-shaped untreated polystyrene plates (Greiner Bio-One, Kremsmünster, Austria) contained a final volume of 100 µL with two-fold serial dilutions of each antibiotic. Microplates were incubated for 16–20 h at 37 °C, with the MIC determined as the lowest concentration of antibiotic that prevented visible growth. A negative control (CAMHB only) and a positive control (bacteria only) were included on each plate. At least three independent assays were conducted for all strains included in this study.

Additionally, the same protocol was applied using CAMHB supplemented with glucose (D(+)-Glucose anhydrous for molecular biology, PanReac AppliChem, Barcelona, Spain), with concentrations ranging from 0 to 1000 mg/dL (corresponding to the maximum value observed in the urinalysis of the dog). The MIC was determined for each antibiotic in the presence of varying concentrations of glucose.

#### 2.3.4. Biofilm Assays

Biofilm biomass quantification and the influence of glucose and antibiotics were evaluated using the crystal violet staining method, as previously described [42,43]. All experiments were performed in sterile 96-well flat-bottomed untreated polystyrene microtiter plates (Greiner Bio-One), using bacterial suspensions of 1 × 10^6^ CFU/mL prepared in unsupplemented Tryptone Soy Broth (TSB, Biokar Diagnostics, Allone, Beauvais, France). Positive controls (bacteria without treatment), negative controls (TSB without inoculum), and *E. coli* ATCC 25922 (reference strain) were included in all assays.

To assess the effect of glucose on total biofilm biomass, glucose concentrations of 0, 62.5, 125, 250, 500, and 1000 mg/dL were tested. The highest concentration (1000 mg/dL) was selected to mimic the maximum glucose level observed in canine urinalysis.

**Figure 1:**
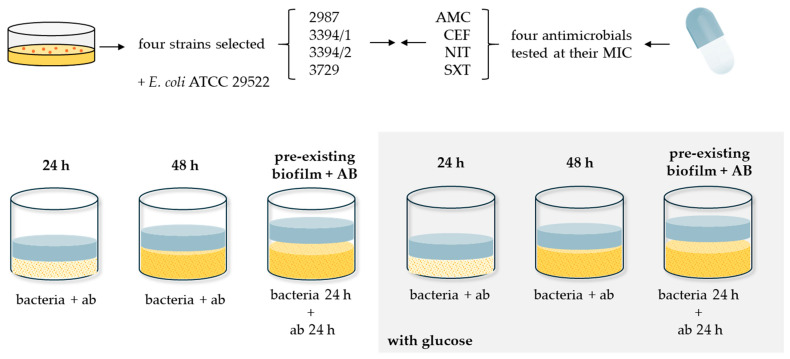
Experimental design used to evaluate the combined effect of glucose and antibiotics on biofilm biomass. Three experimental setups were performed: (i) 24 h exposure—bacterial suspensions were incubated with antibiotics (at their respective MICs) in TSB, with or without glucose (1000 mg/dL), for 24 h at 37 °C; (ii) 48 h exposure—the same design was extended to 48 h of continuous antibiotic exposure; and (iii) pre-existing biofilm + AB—bacterial suspensions were first incubated for 24 h (with or without glucose) to allow biofilm formation, followed by the addition of antibiotics at their MICs and further incubation for 24 h. Biofilm biomass was quantified at the end of each experiment. A total of 120 assay combinations were tested, each performed in triplicate, resulting in a minimum of 360 biofilm quantification assays.

The combined effect of glucose and antibiotics on biofilm biomass was evaluated using four antibiotics (AMC, CEF, NIT, and SXT) fixed at their MIC, which ranged from 8/2 to 4/2 µg/mL for AMC, 16 to 8 µg/mL for CEF, 8 to 1 µg/mL for NIT, and 0.13/2.38 to 0.06/1.19 µg/mL for SXT, depending on the tested isolate. Two conditions were analyzed: (i) the absence of glucose (0 mg/dL) and (ii) the presence of glucose at 1000 mg/dL. Three experimental setups were conducted (Figure 1):

- Exposure for 24 h: Bacterial suspensions in TSB with or without 1000 mg/dL glucose were incubated with antibiotics at their MIC for 24 h at 37 °C.
- Exposure for 48 h: The same experimental design was extended to 48 h of antibiotic exposure, followed by biomass quantification.
- Pre-existing biofilm + AB: Biofilms were first formed by incubating bacterial suspensions with or without 1000 mg/dL glucose for 24 h at 37 °C. After this period of biofilm establishment, antibiotics at their MIC were added, and incubation continued for an additional 24 h (total incubation time: 48 h). Biofilm biomass was then quantified at the 48 h.

Following incubation, biofilms were heat-fixed at 60 °C for 1 h, stained with 0.5% (*v*/*v*) crystal violet (Química Clínica Aplicada, Amposta, Spain) for 5 min, and resolubilized with 33% (*v*/*v*) acetic acid (Acetic Acid Glacial, AppliChem, Darmstadt, Germany). Absorbance was measured at 570 nm using a microplate reader (Thermo Scientific Multiskan^®^ FC, Thermo Fisher Scientific, Waltham, MA, USA), with background absorbance (TSB without inoculum) subtracted from each sample. At least three independent assays were conducted for all experimental conditions.

Also, two additional analyses were performed in all clinical case isolates (excluding *E. coli* ATCC 25922): (i) the total biomass quantified across all four antibiotics, with and without glucose, for each setup (24 h, 48 h, and pre-existing biofilm + AB), and (ii) the cumulative effect of each antibiotic across all bacterial strains tested, with and without glucose, in each experimental condition.

Statistical differences in biofilms formation among isolates, under different glucose concentrations and antibiotic treatments, were assessed using the Kruskal–Wallis test in IBM SPSS Statistics (version 29), with the significance level set at α = 0.05.

#### 2.3.5. MBIC Assays

The MBIC was determined with slight modifications to established protocols [44]. MBIC was evaluated under two conditions: (i) in the presence of each antibiotic alone and (ii) in the simultaneous presence of glucose (1000 mg/dL) alongside each antibiotic. In brief, bacterial suspensions of 1 × 10^6^ CFU/mL in TSB, with or without glucose, were incubated at 37 °C for 24 h in sterile 96-well flat-bottomed untreated polystyrene microtiter plates. Positive and negative controls were also included, as well as *E. coli* ATCC 25922. Biofilms were then washed twice with sterile saline to remove planktonic cells and exposed to increasing antibiotic concentrations (AMC, CEF, NIT, and SXT), ranging from the MIC to at least 256× MIC. This upper limit was selected based on compound solubility, clinical relevance, and methodological feasibility. For NIT and SXT, dimethyl sulfoxide (DMSO, Alfa Aesar, Kandel, German) was used as a solvent, with in-test concentrations kept below 1% [45,46]. Optical density at 620 nm (OD_620_) was measured immediately before antibiotic addition (baseline) and after an additional 24 h of incubation at 37 °C. MBIC was defined as the lowest antibiotic concentration preventing bacterial proliferation, indicated by no increase or a ≤10% increase in OD_620_ compared to the baseline reading [44,47]. Three independent assays were performed per strain, with triplicates for each condition.

## 3. Results

### 3.1. Clinical Case Description

**Figure 2:**
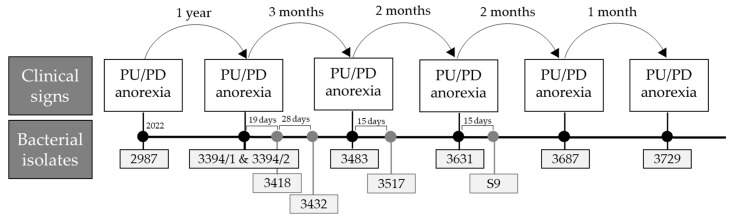
Timeline of clinical signs and sample collection with corresponding bacterial isolate numbers in the selected clinical case.

**Figure 3:**
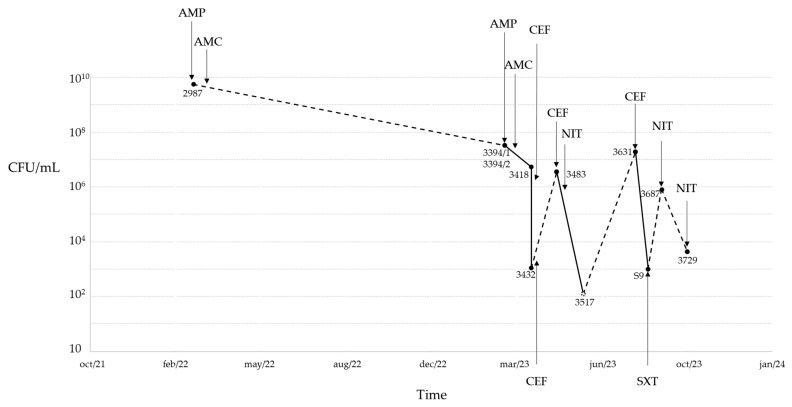
Clinical case timeline and corresponding colony-forming units per milliliters (CFU/mL), isolate detection (2987, 3394/1, 3394/2, 3418, 3432, 3483, 3517, 3631, S9, 3687, and 3729), and antibiotic therapy. The dashed line represents distinct UTI episodes. AMP, ampicillin; AMC, amoxicillin–clavulanate; CEF, cefalexin; NIT, nitrofurantoin; SXT, trimethoprim/sulfamethoxazole.

A detailed clinical timeline summarizing diagnoses, clinical signs, urinary glucose concentrations, bacterial isolates, and administered antibiotic therapies (including drug, dose, and duration) is presented in Table 1 and Figure 2 and Figure 3.

The selected case involved an 11-year-old female Labrador Retriever with a medical history of chronic conditions, including dilated cardiomyopathy, hypothyroidism, and type 1 diabetes mellitus.

The first UTI episode occurred in March 2022, with the dog presenting symptoms of polyuria/polydipsia (PU/PD) and anorexia and marked hyperglucosuria (glucose ≥ 1000 mg/dL—the upper detection limit of the urinary strip test (Combur-Test^®^, Roche, Basel, Switzerland). Treatment with AMP (22 mg/kg, intravenous (IV), TID) was initiated and urine culture confirmed *E. coli* infection (isolate 2987). Based on antimicrobial susceptibility results, therapy was adjusted to AMC (*per os* (PO), 20 mg/kg, BID) for 10 days.

Over the following 18 months, the dog experienced recurrent UTI episodes, typically associated with PU/PD and hyperglycosuria (glucose ≥ 1000 mg/dL). Each episode required urine culture and antibiotic therapy adjustment.

In March 2023, a second UTI episode was diagnosed, and AMP (22 mg/kg, IV, TID) was reintroduced. The treatment was subsequently switched to AMC (20 mg/kg, PO, BID), as oral AMP was not available. Two *E. coli* strains with different resistance profiles (3394/1 and 3394/2; 3.2 × 10^7^ and 6.7 × 10^5^ CFU/mL, respectively) were isolated. Both strains were susceptible to AMC, and the treatment was maintained accordingly. Five days post-therapy, a control culture yielded *E. coli* 3418 (5.3 × 10^6^ CFU/mL), prompting a switch to CEF (20 mg/kg, PO, BID) and Cystocure forte^®^ (100 mg/kg, PO, BID) for 28 days. At the end of this treatment, *E. coli* 3432 (1.1 × 10^3^ CFU/mL) was still detected, prompting an additional 28-day treatment cycle. Following the completion of this final course, antibiotic therapy was discontinued due to the animal’s clinical improvement.

After 15 days (May 2023), clinical signs recurred. CEF (20 mg/kg, PO, BID) was administered for 10 days but isolate 3483 (3.5 × 10^6^ CFU/mL) led to a change to NIT (10 mg/kg, PO, SID) and Uripac^®^ (1 mg/kg, PO, SID) for 14 days. Fifteen days later, *E. coli* 3517 was detected (125 CFU/mL) only by FISH. Treatment was discontinued due to clinical improvement.

After 2 months, July 2023, *E. coli* 3631 (1.9 × 10^7^ CFU/mL) was isolated. After 15 days of CEF, cultures remained positive (isolate S9), leading to a switch to SXT (15 mg/kg, PO, BID) for 8 days.

Nineteen days later (September 2023), a routine check-up revealed glucose ≥ 1000 mg/dL; *E. coli* 3687 (8 × 10^5^ CFU/mL) was isolated and NIT was prescribed (PO, BID, 14 days).

One month later (October 2023), clinical signs recurred. NIT (100 mg, PO, TID, 14 days) was reintroduced, and *E. coli* 3729— exhibiting a distinct antimicrobial profile compared to previous strains, was recovered. However, due to clinical deterioration, characterized by ulcerative necrotic dermatitis, decompensated diabetes mellitus, PU/PD, prostration, keratoconjunctivitis sicca, and suspected pyelonephritis, euthanasia was performed five days later.

### 3.2. E. coli Isolates

#### 3.2.1. Phenotypic and Biochemical Characterization

All isolates were subjected to phenotypic evaluation and biochemical testing. Each one displayed Gram-negative staining, tested negative for oxidase activity, and exhibited glucose fermentation and indole production, while also testing negative for urease. All strains were positive for both lactose and glucose fermentation.

Regarding phenotypic appearance, isolate 3394/2 was particularly noteworthy for its pronounced mucoid characteristics, featuring larger colonies that differed significantly from the remaining isolates, which displayed small, round, well-defined, and non-mucoid colonies (Figure S1).

#### 3.2.2. Kirby–Bauer Method

Antimicrobial susceptibility testing was conducted using the Kirby–Bauer method on all isolates (Table S1).

Resistance to AMP was observed in 8 out of 10 isolates (80%), while resistance to the fluoroquinolone class was detected in 9 out of 10 isolates (90%).

Furthermore, the antimicrobial susceptibility profiles of isolates 2987, 3394/2, 3418, 3432, 3483, 3517, 3631, S9, and 3687 align closely, all showing resistance to both AMP and fluoroquinolones. Isolate 3394/1 demonstrated resistance only to the fluoroquinolone class, while isolate 3729 was susceptible to all tested antibiotics.

#### 3.2.3. WGS Characterization of the Selected *E. coli* Isolates

##### Genomic Profiles

The isolates 2987, 3394/1, 3394/2, 3418, 3432, and 3729 underwent WGS analysis (Table S2).

All isolates belong to the same pathotype, serotype, MLST type, ST clonal complex, cgMLST cluster, and phylogroup: UPEC, O75:H5, 1193, 14, 161277, and B2, respectively.

Isolates 2987, 3394/2, 3418, and 3432 exhibited identical genomic profiles, including acquired genes associated with penicillin resistance (*bla*_TEM-1D_) and resistance to hydrogen peroxide (*sitABCD*) and quinolones resistance due to mutations in the quinolone resistance-determining regions (QRDR) (*gyrA*:p.D87N, p.S83L; *parC*:p.S80I; *par*E:p.L416F). Isolate 3394/1 only showed resistance to hydrogen peroxide and QRDR. These results were in accordance with the phenotypic resistance profile. Strain 3729 showed acquired genes associated with hydrogen peroxide, aminoglycoside (*aph*(*6*)*Id*, *aph*(*3″*)*Ib*), macrolide (*mph*(*A*)), sulfonamides (*sul2*), tetracyclines (*tet*(*B*)), trimethroprim (*dfrA17*) resistances, and QRDR.

All isolates carried the same set of virulence genes, including *fyuA*, *pap*, *vat*, *usp*, *yfcV*, *usp*, *iutA*, *fimH*, and *ompT*. The *senB* gene was present in all isolates except 3394/1, while the *sitA* gene was identified in isolates 3394/1, 3394/2, 3418, and 3729. The *cea* gene was only found in strain 3729.

Additionally, all strains shared the *fimH64* allele, which encodes the type-1 fimbriae adhesin.

In terms of plasmid content, all strains carried the Col(BS512), ColpEC648, IncFIA, and IncFIB plasmids, except for strain 3729, which lacked the ColpEC648 plasmid, and strain 3394/1, which only harbored the Col(BS512) and ColpEC648 plasmids.

##### Phylogenetic Relationship of the Selected *E. coli* Isolates

**Figure 4:**
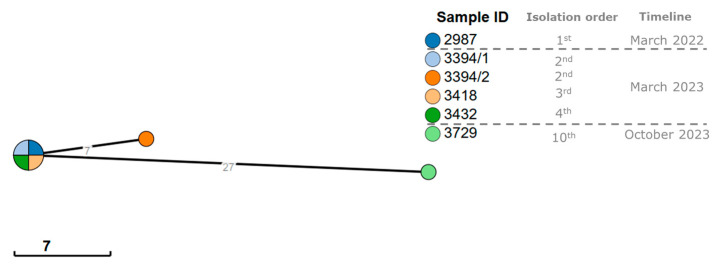
Phylogenetic analysis (minimum spanning tree) of the six *E. coli* isolates using the cgMLST V1 + HierCC V1 scheme with 2513 loci available on the Enterobase platform. The numbers shown on the branches represent allelic differences between the isolates. Clusters were formed with ≤7 allelic differences between isolates and the size of the clusters is proportional to the number of isolates included.

The comparative analysis of the selected isolates using hierarchical clustering with a cut-off ≤ 7 allelic differences [48,49] identified one cluster, comprising isolates 2987, 3394/1, 3418, and 3432 (Figure 4), indicating a high degree of phylogenetic relatedness. The isolates 3394/2 (from a coinfection in the second UTI episode) and 3729 (recovered in the last episode) exhibited 7 and 27 allelic differences, respectively, from this cluster, showing no close phylogenetic relation to the remaining isolates.

### 3.3. Impact of Glucose and Antibiotics in the Selected Strains

Among the six isolates subjected to genomic analysis, 2987, 3394/2, and 3729 were selected for in vitro assays to evaluate the influence of glucose and antibiotics. Isolates 3394/1 was also included, as it was isolated in co-infection with isolate 3394/2. Additionally, *E. coli* ATCC 25922 was used as a reference strain. This selection enabled a focused investigation of the effects of glucose and antibiotics on MIC, MBIC, and biofilm production.

#### 3.3.1. MIC Determination

The MIC was determined for the selected bacteria (2987, 3394/1, 3394/2, 3729, and *E. coli* ATCC 25922, Figure S1) and the antibiotics (AMC, CEF, NIT, and SXT) alone, as well as for the combinations of glucose with antibiotics (Table 2). The MIC values confirmed the results obtained through the Kirby–Bauer method, where all strains were sensitive to the tested antibiotics. In particular, MICs ranged from 4/2 to 8/4 µg/mL for AMC, from 8 to 16 µg/mL for CEF, from 1 to 8 µg/mL for NIT, and from 0.06/1.19 to 0.13/2.38 µg/mL for SX, all below the established EUCAST resistance breakpoints. When evaluating the MIC with different antibiotics and varying concentrations of glucose, the MIC remains consistent compared to the MIC with antibiotics alone. This phenomenon occurs across all strains and with all antibiotics.

#### 3.3.2. Biofilm Biomass

**Figure 5:**
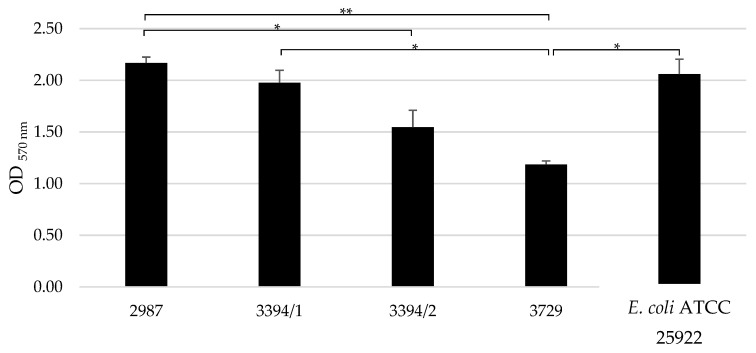
Quantification of biofilm biomass in the selected *E. coli* isolates. Data are shown as mean ± SD of three independent experiments. Statistically significant differences were noted with * (*p* < 0.05) or ** (*p* < 0.01).

The biofilm biomass of the selected strains was quantified using the crystal violet method (Figure 5). Significant differences in biofilm biomass were observed in the following pairs: 2987 and 3394/2, 2987 and 3729, 3394/1 and 3729, and 3729 and *E. coli* ATCC 25922. The selected bacteria displayed heterogeneous biofilm production.

Biofilm biomass quantification was evaluated under varying concentrations of glucose (0 to 1000 mg/dL) (Table 3 and Table S3). A statistically significant increase in biofilm biomass was observed at higher glucose concentrations (1000 mg/dL) compared to the glucose-free condition across all strains, including *E. coli* ATCC 25922.

**Figure 6:**
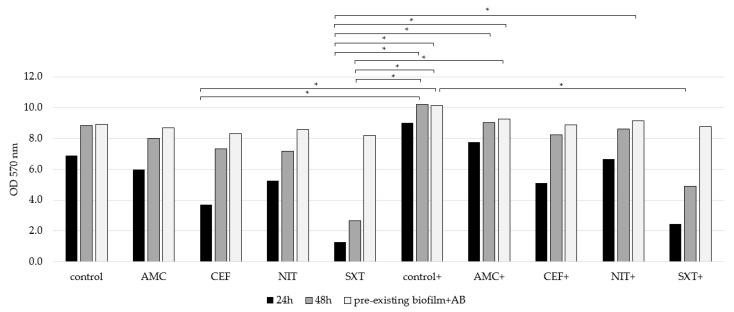
Total biomass quantified across in all clinical case isolates under different conditions: in the absence (control) and presence of glucose (control+). Antibiotics were tested both in the absence of glucose (AMC, CEF, NIT, and SXT) and in its presence (AMC+, CEF+, NIT+, and SXT+). Measurements were performed in three setups: 24 h, 48 h, and pre-formed biofilm plus antibiotic (pre-formed biofilm + AB). Data are shown as means ± SD of three independent experiments. Statistically significant differences were noted with * (*p* < 0.05).

**Figure 7:**
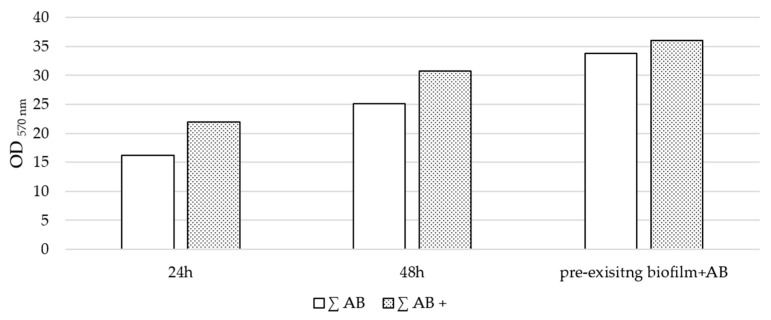
Total biomass quantified across all four antibiotics, with and without glucose (∑AB+, ∑AB, respectively), for each setup (24 h, 48 h, and pre-formed biofilm + AB) in all clinical case isolates.

Additionally, biofilm biomass quantification was assessed in the presence of the highest glucose concentration (1000 mg/dL) combined with the MIC of each tested antibiotic (AMC, CEF, NIT, and SXT—Table 4) under three experimental setups: (1) 24 h exposure, (2) 48 h exposure, and (3) antibiotic exposure after biofilm establishment (pre-existing biofilm + AB) (Figure 6, Figure 7 and Figure S2).

In the first setup (24 h), the significant inhibition of biofilm production was observed in all tested strains across all antibiotics, with varying levels of efficacy: SXT demonstrated the highest inhibition, followed by CEF, NIT, and AMC. In the second setup (48 h), the inhibition pattern remained consistent with that observed at 24 h, with SXT again showing the greatest reduction, followed by CEF, NIT, and AMC. However, the overall inhibition of biofilm production was reduced compared to the first setup. Similarly, in the third setup, the inhibition pattern was comparable (with SXT leading, followed by CEF, NIT, and AMC), but the level of inhibition was lower than that observed in the second setup. Although antibiotic exposure in glucose-supplemented conditions resulted in reduced biomass levels, the magnitude of these reductions was less pronounced compared to conditions without glucose. While glucose did not alter the efficacy of antibiotics across all experimental setups, it contributed to an overall increase in total biomass, although these differences were not statistically significant (Figure 6).

#### 3.3.3. MBIC Determination

The MBIC was determined in both the presence (1000 mg/dL) and absence of glucose (Table 5).

MBIC values could only be determined in the absence of glucose for AMC and CEF, and only for strains 3394/2, 3729, and *E. coli* ATCC 25922. For AMC, the MBIC ranged from 8 × MIC (strain 3729) to 16 × MIC (*E. coli* ATCC 25922). For CEF, MBIC values were markedly higher, reaching 256 × MIC for strains 3729 and *E coli* ATCC 25922, and 512 × MIC for strain 3394/2. In all cases where MBIC values were measurable, the MBIC in the presence of glucose was higher than the MBIC in the absence of glucose.

## 4. Discussion

Urinary tract infections (UTIs) are a common and recurrent disease in both human and veterinary medicine, often complicated by the presence of biofilm-producing and antibiotic-resistant bacterial strains [15,50,51,52].

This study examined a clinical case involving an 11-year-old female Labrador Retriever with recurrent UTIs. The aim was to analyze the antimicrobial resistance development and the spectrum of biofilm production capacities of *E. coli* isolates obtained during the time-course of the disease, offering deeper insights into the persistence and recurrence of UTIs in complex cases and providing clinically relevant data to support more informed treatment decisions. For this reason, the focus was on endpoints of higher levels of biological organization with clinical relevance, such as antimicrobial resistance and biofilm formation. In this context, information provided by genotypes was complementary to phenotypic observations of resistance.

Regarding antimicrobial susceptibility, nine out of the eleven isolates exhibited the same antimicrobial resistance profile, being resistant to ampicillin and fluoroquinolones, which are among the most commonly prescribed antimicrobial classes in veterinary medicine in Portugal, ranking first and fourth, respectively [53].

One intriguing finding was the phenotypic and genotypic differences between isolates 3394/1 and 3394/2, both derived from the same sample. For instance, isolate 3394/2, which was more mucoid and produced a weaker biofilm, was identical in terms of resistance and genomics to isolate 2987. The mucoid appearance likely reflects the increased production of exopolysaccharides such as alginate or capsule, which can confer protection through a thick extracellular matrix but do not necessarily correlate with greater biofilm biomass or stronger attachment [54,55,56]. In contrast, isolate 3394/1, taken from the same sample, produced a substantial amount of biofilm but did not demonstrate antimicrobial resistance. These findings support the hypothesis of a possible trade-off between antimicrobial resistance and biofilm formation, where some isolates “invest” in biofilm production as a tolerance and persistence strategy, while others rely on acquired genetic resistance mechanisms to survive under antimicrobial pressure. The clinical management becomes substantially more challenging when both capabilities coexist in the same site of infection, as previously reported in cases of polymicrobial infections, where different bacterial strains or species dramatically affect the clinical efficiency of antibiotics, due to the establishment of “antibiotic resistant environments” [57,58]. Interestingly, isolate 3729 represented a distinct phenotype: it displayed both low biofilm production and full susceptibility to all tested antibiotics. For example, this isolate harbors unique virulence genes such as *cea*, which may contribute to persistence through mechanisms other than biofilm production, such as the suppression of epithelial exfoliation and the enhanced colonization of the urinary tract [59]. Moreover, the presence of such virulence factors could make this isolate particularly advantageous within mixed-strain infections, complementing highly resistant or strong biofilm-producing bacteria and contributing to the establishment of highly recalcitrant polymicrobial consortia. Thus, the bacterial strategies for survival and persistence in the host appear to be multifaceted and not exclusively reliant on either biofilm production or antimicrobial resistance.

Genomic analysis revealed that all isolates in this study belonged to ST1193, the B2 phylogenetic group, and the O75 serogroup. ST1193, derived from ST clonal complex 14, is recognized as a high-risk, multidrug-resistant clone, with a pathogenic probability ≥92% in humans, commonly linked to recurrent UTIs and bloodstream infections [52,60]. Since its emergence, this lineage has been increasingly reported worldwide, including in Australia, China, Korea, Europe, and the USA, where it is recognized as the second most prevalent clone among fluoroquinolone and cephalosporin resistant *E. coli* isolates [52,60,61]. Although primarily described in humans, evidence also suggests that companion animals likely serve as spillover hosts rather than primary reservoirs for this lineage [62,63]. Moreover, this clone is mimicking the successful global spread of ST131 [60,63]. ST1193 is derived from ST clonal complex 14, is part of phylogenetic group B2, and is characterized by the O75 serogroup [60]. Unlike ST131, ST1193 is associated with *fimH64* allele [63]. Notably, the detection of ST1193 in companion animals mirrors the global trend observed in humans, emphasizing the potential cross-species transmission and its significance for public health [64]. Therefore, further research is warranted to elucidate the epidemiology, transmission pathways, and clinical impact of this ST, thereby contributing to a more comprehensive understanding of its role in antimicrobial resistance.

Also, phylogroup B2 has been linked to enhanced biofilm production in humans, with its prevalence rising from 20% in 2014 to 83% in 2020, paralleling an increase in UPEC infections [65,66]. This expansion underscores the virulence potential of B2 strains, driven by their capacity for tissue adhesion and biofilm formation [66].

All strains in this study carried virulence genes that supported their classification as UPEC, including *chuA*, *fyuA*, *pap*, *vat*, *usp*, and *yfcV* [67]. These genes, along with *ompT*, *iutA* and *fimH*, are closely associated with UPEC pathogenesis, enhancing the ability of *E. coli* to efficiently colonize the urinary tract [38,68,69,70,71]. Furthermore, strains expressing P-fimbriae, encoded by the *pap* gene, are more likely to cause severe UTIs, including pyelonephritis. The *pap* gene is frequently used as a marker to identify highly virulent UPEC strains [66]. Interestingly, strain 3729 was the only isolate found to carry the *cea* gene, which is associated with the suppression of epithelial exfoliation and the enhanced colonization of the urogenital tract [59]. This may be related to its lower biomass production (reduced biofilm formation capacity) compared to other strains, suggesting a compensatory reliance on a broader set of virulence genes to maintain colonization efficiency.

Phylogenetic analysis revealed the persistence of a single *E. coli* strain during most infection episodes, with the exception of two isolates: one recovered from a coinfection in the second UTI episode, and the other from the final episode. This finding is supported by the consistent antimicrobial resistance profiles observed among the isolates, suggesting persistence or reinfection by a dominant strain [72]. These results may suggest the presence of a bacterial reservoir within the dog, facilitating the reseeding of the urinary tract and contributing to recurrent infections. Possible reservoirs include the gastrointestinal tract, where the strain may persist as part of the commensal microbiota, as well as intracellular bacterial communities or biofilm-associated populations in the urinary tract [73,74,75,76,77]. The survival of persister cells may further contribute to the chronicity and recurrence of infection despite apparently effective antimicrobial treatment [78,79]. Previous studies have shown that 80% of ST1193 isolates are able to produce moderate to strong biofilms, despite exhibiting only moderate adherence and low invasion capacity [80]. In the present study, total biomass quantification among the selected *E. coli* strains was heterogeneous, with a statistically significant increase observed at high glucose concentrations. This observation is particularly relevant for diabetic patients, as elevated glucose levels in urine are known to significantly enhance biofilm formation [81]. Interestingly, previous research has demonstrated that the non-biofilm-producing clinical isolates of *Staphylococcus* spp. can become biofilm producers in the presence of sodium chloride and high glucose concentrations, regardless of the presence or absence of the *ica* operon [82]. A similar effect has been reported for *P. aeruginosa*, where glucose availability has promoted biofilm formation [83]. Glucosuria typically results from hyperglycemia and is a hallmark of diabetes mellitus; however, it is not exclusive to this condition [84]. In humans, significant glucosuria, defined as urinary glucose concentrations above 15 mg/dL, usually occurs when blood glucose exceeds 180 mg/dL [85]. Although fewer studies address this threshold in veterinary medicine, glucosuria in dogs generally develops when blood glucose exceeds approximately 200 mg/dL [86]. In veterinary clinical testing, urine glucose readings typically range from ≤75 mg/dL to >750 mg/dL, with samples containing values above 75 mg/dL considered positive for glucosuria across different analytical techniques [87]. These urinary glucose concentrations are commonly observed in diabetic animals, thereby creating an environment that may favor the biofilm-mediated persistence of pathogens such as *E. coli*.

Regarding total biomass quantification, sulfamethoxazole–trimethoprim showed the strongest inhibitory effect among the antibiotics tested (AMC, CEF, and NIT), consistently promoting the greatest reductions across all tested strains and experimental setups (24 h, 48 h, and pre-existing biofilm + 24 h antibiotic exposure). This can be attributed to SXT synergistic mechanism of action, which involves the inhibition of tetrahydrofolate synthesis, essential for nucleotide production and biofilm formation, particularly in strains such as *Acinetobacter baumanii* ATCC 17978 [88]. In contrast, amoxicillin–clavulanate exhibited the weakest biofilm inhibition in the present study. Although it is considered as an effective antibiofilm agent in certain clinical situations, such as chronic rhinosinusitis with nasal polyposis, its efficacy in inhibiting biofilm varies depending on the bacterial species and the extent of biofilm formation [89].

This study suggests that although glucose does not directly affect the activity of the antibiotics, it contributes to an overall increase in total biofilm biomass. This may be due to the increased baseline levels of biofilm biomass formed in glucose-supplemented environments, which could enhance biofilm-mediated antibiotic tolerance and reduce antibiotic susceptibility. For instance, high glucose availability has been shown to promote the production of extracellular polymeric substances (EPS) in *P. aeruginosa*, leading to thicker, more robust biofilms with increased antibiotic tolerance [83]. Moreover, previous studies suggested that glucose can repress the cAMP–CRP system (responsible for modulating stress responses, virulence, and drug susceptibility) and can also promote increased ATP production and capsular polysaccharide synthesis, both of which may further support bacterial survival under antibiotic pressure [90,91,92,93]. Although species-specific differences exist, similar glucose-induced biofilm enhancement mechanisms may also be relevant in *E. coli*, potentially reducing antibiotic susceptibility and contributing to treatment failure under hyperglycemic or glycosuric conditions.

Hyperosmotic stress, such as that induced by high glucose concentrations in urine, profoundly alters bacterial physiology by reducing cell volume and slowing bacterial growth, ultimately promoting the transition from a planktonic to a biofilm-associated phenotype [94]. While glucose may support bacterial growth by acting as a nutrient, extreme levels of this sugar can trigger biofilm production, potentially contributing to therapeutic failure [95,96]. Previous studies demonstrated that the effects of glucose in human urine on UPEC strains acted not only as a virulence-enhancing nutrient but also induced physiological adaptations that may compromise treatment outcomes [97,98]. Notably, hyperosmotic shock activates the biofilm-dependent modulation via the RcsCDB sensory system, which regulates extracellular components critical for biofilm formation [99]. In addition, high glucose concentrations may also affect the expression of virulence genes through the cAMP-CRP pathway, modulating stress responses and promoting the synthesis of extracellular polysaccharides, which are key components of the biofilm matrix [100]. Nonetheless, further transcriptomic studies (e.g., RNA sequencing) under hyperglycemic conditions would be valuable to clarify how glucose modulates the regulation of virulence and biofilm-related genes, thereby clarifying the molecular mechanisms supporting these phenotypic observations.

At 48 h, a slight decrease in biofilm biomass was observed across all conditions, likely due to the combined effects of antibiotic degradation over time and the adaptive responses of bacterial populations within the biofilm [101,102].

Also, in the third experimental setup, where antibiotics were applied after biofilm establishment, the inhibitory effects were even less pronounced. Pre-formed biofilms may represent a more challenging scenario compared to those that develop in the continuous presence of antibiotics. This reduced efficacy is consistent with the increased tolerance observed in mature biofilms, which is often driven by physiological adaptations, such as the formation of persister cells and the activation of stress responses [103,104]. Biofilm-associated bacteria can enter a dormant state, which enhances survival against antibiotic treatment, while stress responses, like the SOS and stringent pathways, further promote persistence and complicate eradication efforts [104,105]. Among the tested conditions, this final setup most closely mimics the in vivo situation, where biofilms are generally established prior to the initiation of antimicrobial treatment. Notably, this assay suggests that, even in the absence of diabetes mellitus, treating biofilm-associated infections would still pose significant challenges. Once a biofilm is established, antibiotic intervention alone may no longer be effective. Recent exposure to multiple antibiotics within the past 30 days, along with a clinical history of recurrent UTIs, has been positively associated with enhanced biofilm formation [57].

According to our results, glucose did not affect the minimum inhibitory concentrations of the antibiotics tested. However, it influenced the minimum biofilm inhibitory concentrations. Notably, when MBIC could be determined, the values were higher in the presence of both glucose and antibiotics, suggesting that the presence of glucose may enhance bacterial biofilm production in response to antibiotic exposure, thereby increasing their resistance to these therapeutic agents. In fact, biofilm formation significantly promotes bacterial survival by limiting antibiotic penetration and preventing the accumulation of bactericidal concentrations within the biofilm matrix [105,106]. As a result, biofilm-associated cells can be 100 to 1000 times more tolerant to antimicrobials than their planktonic counterparts [107]. Clinical studies have shown that *Staphylococcus aureus* biofilms from diabetic foot infections showed MBIC values up to 1000 times greater than corresponding MIC [108]. In these clinical cases, MBIC determination might be more valuable than traditional MIC assays, as it provides a more accurate assessment of antibiotic efficacy.

Given the antibiotic susceptibility results and the limited effectiveness of most antibiotics against established biofilms, therapeutic options become particularly challenging in diabetic animals with recurrent UTIs. In this context, SXT stands out not only for its consistent antibiofilm activity observed in this study, even against mature biofilms, but also for its classification as a first-line treatment by the European Medicines Agency (EMA) [27]. However, as demonstrated in this clinical case, treatment inevitably becomes a daunting challenge whenever acquired resistance, antimicrobial tolerance, and ecological conditions favorable to biofilm formation coexist. Moreover, in vivo effectiveness may be limited by host factors such as immune status, pharmacokinetics, and drug metabolism, and tissue distribution, which can reduce antibiotic concentrations at the site of infection and allow biofilm persistence despite in vitro susceptibility [109]. Accordingly, the clinical management of UTIs should incorporate strict control of glucosuria as a key component of treatment, underscoring the importance of addressing both the primary disease (e.g., diabetes mellitus) and its secondary complications in an integrated manner.

## 5. Conclusions

This study highlights the complexity of managing recurrent UTIs in diabetic animals, where biofilm formation and antimicrobial resistance significantly contribute to persistence and recurrence. WGS revealed that the *E. coli* isolates belonged to the multidrug-resistant ST1193 lineage, a globally emerging clone associated with chronic and recurrent infections. Phylogenetic analysis confirmed the clonal persistence of the same strain across most infection episodes, suggesting either persistence or reinfection by a dominant strain. Increased glucose levels significantly enhance biofilm formation and reduce antibiotic efficacy, contributing to the recurrence and persistence of infections. Sulfamethoxazole–trimethoprim showed strong antibiofilm activity, making it a promising treatment for recurrent UTIs in diabetic animals. These findings emphasize the need for targeted strategies that address both antimicrobial resistance and biofilm-associated tolerance, particularly in diabetic patients. Hence, this work underscores the need for improved surveillance, the effective management of glucose levels, and alternative therapeutic approaches to address biofilm-associated infections.

## Figures and Tables

**Table 1:** Clinical case timeline, with corresponding diagnoses, clinical signs, glucose concentrations, antibiotherapy (including antibiotic, dose, and duration), and bacterial isolates recorded at each timepoint.

Time	Pathology	Clinical Signs	Glucose Concentration	Antibiotherapy			Isolates
				Antibiotic	Dosage	Duration	
March 2022	UTI	PU/PD, anorexia	>1000 mg/dL	AMP * AMC	22 mg/kg 20 mg/kg	2 days 10 days	2987
March 2023	UTI	PU/PD, anorexia	>1000 mg/dL	AMP * AMC CEF	22 mg/kg 20 mg/kg 20 mg/kg	2 days 14 days 28 days	3394/1 and 3394/2 3418 3432
May 2023	UTI	PU/PD, anorexia	>1000 mg/dL	CEF * NIT	20 mg/kg 10 mg/kg	10 days 14 days	3483 3517
July 2023	UTI	PU/PD, anorexia	>1000 mg/dL	CEF * SXT	20 mg/kg 15 mg/kg	15 days 8 days	3631 S9
September 2023	UTI	PU/PD, anorexia	>1000 mg/dL	NIT *	10 mg/kg	14 days	3687
October 2023	UTI	PU/PD, anorexia	>1000 mg/dL	NIT *	10 mg/kg	14 days	3729

UTI, urinary tract infection; PU/PD, polyuria/polydipsia; AMP, ampicillin; AMC, amoxicillin-clavulanate; CEF, cefalexin; SXT, trimethoprim/sulfamethoxazole; NIT, nitrofurantoin; *, empirical antibiotherapy.

**Table 2:** Minimum inhibitory concentrations (MIC, in µg/mL) of *E. coli* strains against antibiotics (AMC, AMP, CEF, NIT, and SXT) at different glucose concentrations.

Isolate	Glucose Concentration (mg/dL)	AMC	CEF	NIT	SXT
2987	0	8/4	8	1	0.06/1.19
	62.5	8/4	8	1	0.06/1.19
	125	8/4	8	1	0.06/1.19
	250	8/4	8	1	0.06/1.19
	500	8/4	8	1	0.06/1.19
	1000	8/4	8	1	0.06/1.19
3394/1	0	8/4	16	8	0.13/2.38
	62.5	8/4	16	8	0.13/2.38
	125	8/4	16	8	0.13/2.38
	250	8/4	16	8	0.13/2.38
	500	8/4	16	8	0.13/2.38
	1000	8/4	16	8	0.13/2.38
3394/2	0	8/4	8	8	0.06/1.19
	62.5	8/4	8	8	0.06/1.19
	125	8/4	8	8	0.06/1.19
	250	8/4	8	8	0.06/1.19
	500	8/4	8	8	0.06/1.19
	1000	8/4	8	8	0.06/1.19
3729	0	4/2	16	4	0.13/2.38
	62.5	4/2	16	4	0.13/2.38
	125	4/2	16	4	0.13/2.38
	250	4/2	16	4	0.13/2.38
	500	4/2	16	4	0.13/2.38
	1000	4/2	16	4	0.13/2.38
*E. coli* ATCC 25922	0	8/4	16	4	0.13/2.38
	62.5	8/4	16	4	0.13/2.38
	125	8/4	16	4	0.13/2.38
	250	8/4	16	4	0.13/2.38
	500	8/4	16	4	0.13/2.38
	1000	8/4	16	4	0.13/2.38

AMC, amoxicillin–clavulanic acid (2:1); AMP, ampicillin; CEF, cefalexin; NIT, nitrofurantoin; SXT, trimethoprim/sulfamethoxazole (1:19).

**Table 3:** Effect of different glucose concentrations on biofilm biomass.

Isolate	Glucose Concentration (mg/dL)	Mean OD ± SD	*p*-Value
2987	0	2.113 ± 0.066	
	62.5	2.276 ± 0.268	0.359
	125	2.383 ± 0.165	0.126
	250	2.496 ± 0.055	0.039 *
	500	2.500 ± 0.113	0.027 *
	1000	2.557 ± 0.232	0.027 *
3394/1	0	1.929 ± 0.128	
	62.5	1.979 ± 0.056	0.646
	125	1.995 ± 0.086	0.444
	250	2.008 ± 0.147	0.491
	500	2.125 ± 0.142	0.126
	1000	2.423 ± 0.140	0.006 **
3394/2	0	1.631 ± 0.119	
	62.5	1.782 ± 0.127	0.515
	125	2.086 ± 0.141	0.072
	250	2.099 ± 0.171	0.047 *
	500	2.191 ± 0.150	0.010 *
	1000	2.310 ± 0.169	0.004 **
3729	0	1.188 ± 0.239	
	62.5	1.356 ± 0.074	0.320
	125	1.247 ± 0.103	0.878
	250	1.191 ± 0.111	0.939
	500	1.808 ± 0.087	0.029 *
	1000	1.923 ± 0.135	0.013 *
*E. coli* ATCC 25922	0	2.012 ± 0.304	
	62.5	2.306 ± 0.277	0.359
	125	2.146 ± 0.206	0.646
	250	2.009 ± 0.221	0.818
	500	2.625 ± 0.097	0.012 *
	1000	2.544 ± 0.084	0.039 *

Data are shown as mean ± SD of three independent experiments. Kruskal–Wallis test was applied to identify differences between the absence of glucose and the tested glucose concentrations (62.5, 125, 250, 500, and 1000 mg/mL). * *p* < 0.05, ** *p* < 0.01.

**Table 4:** Minimum inhibitory concentrations (MIC, µg/mL) of *E. coli* strains for antibiotics used in biofilm biomass quantification assays.

Isolate	AMC	CEF	NIT	SXT
2987	8/4	8	1	0.06/1.19
3394/1	8/4	16	8	0.13/2.38
3394/2	8/4	8	8	0.06/1.19
3729	4/2	16	4	0.13/2.38
*E. coli* ATCC 25922	8/4	16	4	0.13/2.38

AMC, amoxicillin–clavulanic acid (2:1); CEF, cefalexin; NIT, nitrofurantoin; SXT, trimethoprim/sulfamethoxazole (1:19).

**Table 5:** MIC and MBIC (with and without glucose) of each selected strain. Concentrations are expressed in µg/mL.

	AMC			CEF			NIT			SXT		
	MIC	MBIC	MBIC (+Glucose)	MIC	MBIC	MBIC (+Glucose)	MIC	MBIC	MBIC (+Glucose)	MIC	MBIC	MBIC (+Glucose)
2987	8/4	>2048/1024 (>256×MIC)	>2048/1024 (>256×MIC)	8	>8192 (>1024×MIC)	>8192 (>1024×MIC)	1	>64 (>64×MIC)	>64 (>64×MIC)	0.06/1.19	>64/1216 (>1024×MIC)	>64/1216 (>1024×MIC)
3394/1	8/4	>2048/1024 (>256×MIC)	>2048/1024 (>256×MIC)	16	>8192 (>512×MIC)	>8192 (>512×MIC)	8	>64 (>8×MIC)	>64 (>8×MIC)	0.13/2.38	>64/1216 (>512×MIC)	>64/1216 (>512×MIC)
3394/2	8/4	>2048/1024 (>256×MIC)	>2048/1024 (>256×MIC)	8	4096 (512×MIC)	>8192 (>1024×MIC)	8	>64 (>8×MIC)	>64 (>8×MIC)	0.06/1.19	>64/1216 (>1024×MIC)	>64/1216 (>1024×MIC)
3729	4/2	32/16 (16×MIC)	>2048/1024 (>512×MIC)	16	4096 (256×MIC)	>8192 (>512×MIC)	4	>64 (>16×MIC)	>64 (>16×MIC)	0.13/2.38	>64/1216 (>512×MIC)	>64/1216 (>512×MIC)
*E. coli* ATCC 25922	8/4	32/16 (8×MIC)	>2048/1024 (>256×MIC)	16	4096 (256×MIC)	>8192 (>512×MIC)	4	>64 (>16×MIC)	>64 (>16×MIC)	0.13/2.38	>64/1216 (>512×MIC)	>64/1216 (>512×MIC)

AMC, amoxicillin–clavulanic acid (2:1); CEF, cefalexin; NIT, nitrofurantoin; SXT, trimethoprim/sulfamethoxazole (1:19).

## Data Availability

The original contributions presented in this study are included in the article and Supplementary Materials. Further inquiries can be directed to the corresponding author.

## Supplementary Materials

The following supporting information can be downloaded at: https://www.mdpi.com/article/10.3390/microorganisms13081946/s1. Figure S1: Phenotypic appearance of selected *E. coli* isolates. Panels (A–E) display the phenotypes of *E. coli* isolates (a) 2987, (b) 3394/1, (c) 3394/2, (d) 3729, and (e) the reference *E. coli* ATCC 25922. Images were captured using the Bio-Rad™ ChemiDoc Imaging System (Bio-Rad, Hercules, CA, USA); Table S1: Antimicrobial susceptibility profile of E. coli strains from the dog across various sampling times; Table S2: Overview of E. coli isolates genomic characterization; Table S3: Summary of Kruskal–Wallis test statistics for the effect of glucose concentrations on biofilm biomass across isolates; Figure S2: Effect of glucose (0 vs. 1000 mg/dL) on biofilm biomass under different antibiotic exposure conditions in five *E. coli* isolates: (a) Isolate 2987; (b) Isolate 3394/1; (c) Isolate 3394/2; (d) Isolate 3729, and (e) *E. coli* ATCC 25922. Biofilms were treated with four antibiotics, namely amoxicillin–clavulanate (AMC), cefalexin (CEF), nitrofurantoin (NIT), and trimethoprim–sulfamethoxazole (SXT), each at their MIC, in the absence (control) or presence of glucose (control+ and antibiotic+), under three experimental setups: (1) 24 h exposure, (2) 48 h exposure, and (3) antibiotic exposure after biofilm establishment (pre-existing biofilm + AB).
