# Supplementary material for: Uropathogenic Escherichia coli in a Diabetic Dog with Recurrent UTIs: Genomic Insights and the Impact of Glucose and Antibiotics on Biofilm Formation
Source: Microorganisms. 2025 Aug 20;13(8):1946. doi: 10.3390/microorganisms13081946 (PMC12388685; doi:10.3390/microorganisms13081946)
Supplement: Supplementary file 1 [file microorganisms-13-01946-s001.zip › microorganisms-3822367-supplementary.pdf]

Supplementary materials

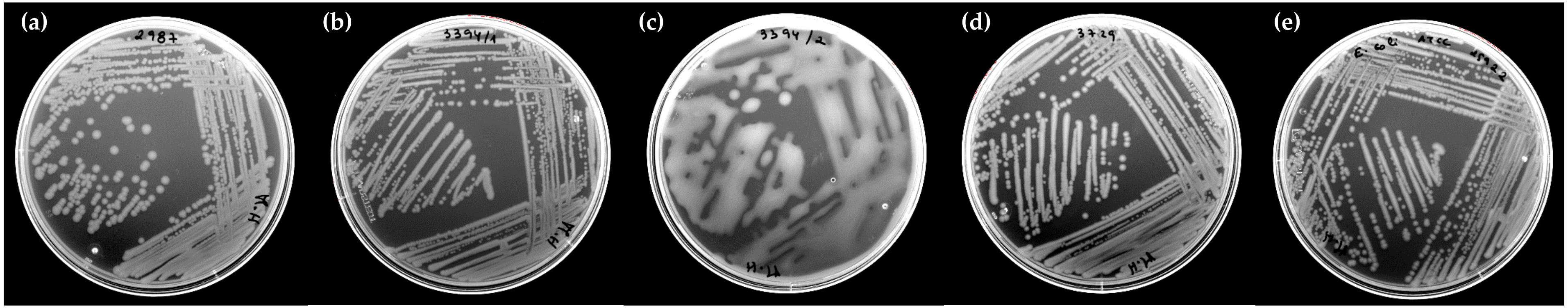

Figure S1. Phenotypic appearance of selected *E. coli* isolates. Panels (A–E) display the phenotypes of *E. coli* isolates (**a**) 2987, (**b**) 3394/1, (**c**) 3394/2, (**d**) 3729, and (**e**) the reference *E. coli* ATCC 25922. Images were captured using the Bio-Rad™ ChemiDoc Imaging System (Bio-Rad, Hercules, CA, USA).

Table S1. Antimicrobial susceptibility profile of *E. coli* strains from the dog across various sampling times.

Antibiotic Class	Antibiotic	2987	3394/1	3394/2	3418	3432	3483	3517	3631	S9	3687	3729
Penicillin	AMP	**R**	S	**R**	**R**	**R**	**R**	**R**	**R**	**R**	**R**	S
	AMC	S	S	S	S	S	S	S	S	S	S	S
Cephalosporins	CAZ	S	S	S	S	S	S	S	S	S	S	S
	CFZ	S	S	S	S	S	S	S	S	S	S	S
	CTX	S	S	S	S	S	S	S	S	S	S	S
	FOX	S	S	S	S	S	S	S	S	S	S	S
Monobactam	ATM	S	S	S	S	S	S	S	S	S	S	S
Aminoglycosides	AMK	S	S	S	S	S	S	S	S	S	S	S
	GEN	S	S	S	S	S	S	S	S	S	S	S

	TOB	S	S	S	S	S	S	S	S	S	S	S
Tetracyclines	DOX	S	S	S	S	S	S	S	S	S	S	S
	TET	S	S	S	S	S	S	S	S	S	S	S
Fluoroquinolones	CIP	**R**	**R**	**R**	**R**	**R**	**R**	**R**	**R**	**R**	**R**	S
	ENR	**R**	**R**	**R**	**R**	**R**	**R**	**R**	**R**	**R**	**R**	S
	MAR	**R**	**R**	**R**	**R**	**R**	**R**	**R**	**R**	**R**	**R**	S
Folate inhibitor	SXT	S	S	S	S	S	S	S	S	S	S	S
Phenicol	CHL	S	S	S	S	S	S	S	S	S	S	S
Nitrofuran	NIT	S	S	S	S	S	S	S	S	S	S	S
Carbapenems	IMP	S	S	S	S	S	S	S	S	S	S	S

R, resistant; I, intermediate; S, susceptible; AMK, amikacin, AMC, amoxicillin/clavulanic acid; AMP, ampicillin; ATM, aztreonam; CAZ, ceftazidime; CHL, chloramphenicol; CIP, ciprofloxacin, CTX, cefotaxime; DOX, doxycycline; ENR, enrofloxacin, FOX, cefoxitin; GEN, gentamicin, IMP, imipenem; CFZ: cefazolin; MAR, marbofloxacin, NIT, nitrofurantoin; SXT, trimethoprim/sulfamethoxazole; TET, tetracycline; TOB, tobramycin.

Table S2. Overview of *E. coli* isolates genomic characterization.

**Strain ID**	**2987**	**3394/1**	**3394/2**	**3418**	**3432**	**3729**
**Pathotype**	UPEC	UPEC	UPEC	UPEC	UPEC	UPEC
**SerotypeFinder**	O75:H5	O75:H5	O75:H5	O75:H5	O75:H5	O75:H5
**ST**	1193	1193	1193	1193	1193	1193
**ST Complex**	14	14	14	14	14	14
**cgMLST (HC5)**	244039	244205	244369	244039	244039	244207
**Phylogroup**	B2	B2	B2	B2	B2	B2
**ResFinder**	bla_{TEM-1B}, *sitABCD*	*sitABCD*	bla_{TEM-1B}, *sitABCD*	bla_{TEM-1B}, *sitABCD*	bla_{TEM-1B}, *sitABCD*	*aph(6)Id, aph(3'')Ib, mph(A), sul2, tet(B), dfrA17, sitABCD*

**QRDR mutations**	*gyrA:*p.D87N, p.S83L*;* *parC:*p.S80I*;* *parE:*p.L416F	*gyrA:*p.D87N, p.S83L*;* *parC:*p.S80I*;* *parE:*p.L416F	*gyrA:*p.D87N, p.S83L*;* *parC:*p.S80I*;* *parE:*p.L416F	*gyrA:*p.D87N, p.S83L*;* *parC:*p.S80I*;* *parE:*p.L416F	*gyrA:*p.D87N, p.S83L*;* *parC:*p.S80I*;* *parE:*p.L416F	*gyrA:*p.D87N, p.S83L*;* *parC:*p.S80I*;* *parE:*p.L416F
**VirulenceFinder**	*AslA, chuA, fimH, fyuA, gad, iha, irp2, iucC, iutA, kpsE, kpsMII_K1, neuC, nlpl, ompT, papA_F43, sat, senB, terC, usp, vat, yehA, yehB, yehC, yehD, yfcV*	*AslA, chuA, fimH, fyuA, gad, iha, irp2, iucC, iutA, kpsE, kpsMII_K1, neuC, nlpl, ompT, papA_F43, sat, sitA, terC, usp, vat, yehA, yehB, yehC, yehD, yfcV*	*AslA, chuA, fimH, fyuA, gad, iha, irp2, iucC, iutA, kpsE, kpsMII_K1, neuC, nlpl, ompT, papA_F43, sat, senB, sitA, terC, usp, vat, yehA, yehB, yehC, yehD, yfcV*	*AslA, chuA, fimH, fyuA, gad, iha, irp2, iucC, iutA, kpsE, kpsMII_K1, neuC, nlpl, ompT, papA_F43, sat, senB, sitA, terC, usp, vat, yehA, yehB, yehC, yehD, yfcV*	*AslA, chuA, fimH, fyuA, gad, iha, irp2, iucC, iutA, kpsE, kpsMII_K1, neuC, nlpl, ompT, papA_F43, sat, senB, terC, usp, vat, yehA, yehB, yehC, yehD, yfcV*	*AslA, cea, chuA, fimH, fyuA, gad, iha, irp2, iucC, iutA, kpsE, kpsMII_K1, neuC, nlpl, ompT, papA_F43, sat, senB, sitA, terC, usp, vat, yehA, yehB, yehC, yehD, yfcV*
**PlasmidFinder**	Col(BS512), ColpEC648, IncFIA, IncFIB(AP001918)	Col(BS512), ColpEC648	Col(BS512), ColpEC648, IncFIA, IncFIB(AP001918)	Col(BS512), ColpEC648, IncFIA, IncFIB(AP001918)	Col(BS512), ColpEC648, IncFIA, IncFIB(AP001918)	Col(BS512), IncFIA, IncFIB(AP001918)
**FimH**	*fimH64*	*fimH64*	*fimH64*	*fimH64*	*fimH64*	*fimH64*

ST, sequence type, cgMLST, core genome MultiLocus Sequence Type, QRDR, quinolone resistance-determining

Table S3. Summary of Kruskal-Wallis test statistics for the effect of glucose concentrations on biofilm biomass across isolates.

	**df**	**H**	**p-value**	**n**
2987	5	7,877	0,163	18
3394/1	5	9,585	0,088	18
3394/2	5	12,371	0,030	18
3729	5	12,937	0,024	18
E. coli ATCC 25922	5	12,522	0,028	18

df, degrees of freedom, n, sample size

(a)

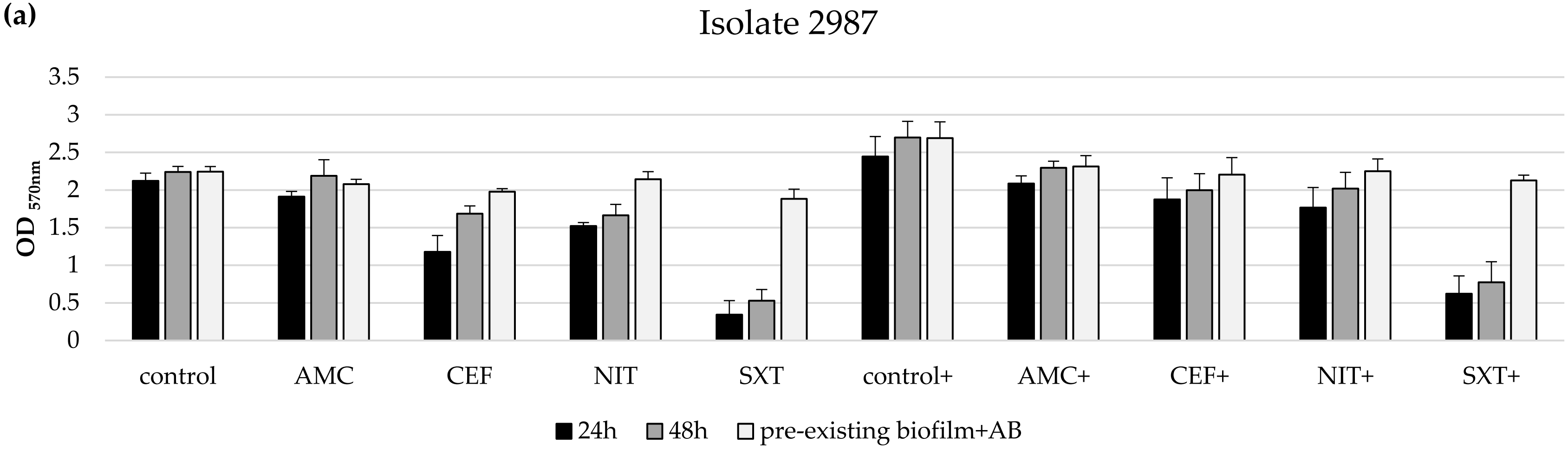

(b)

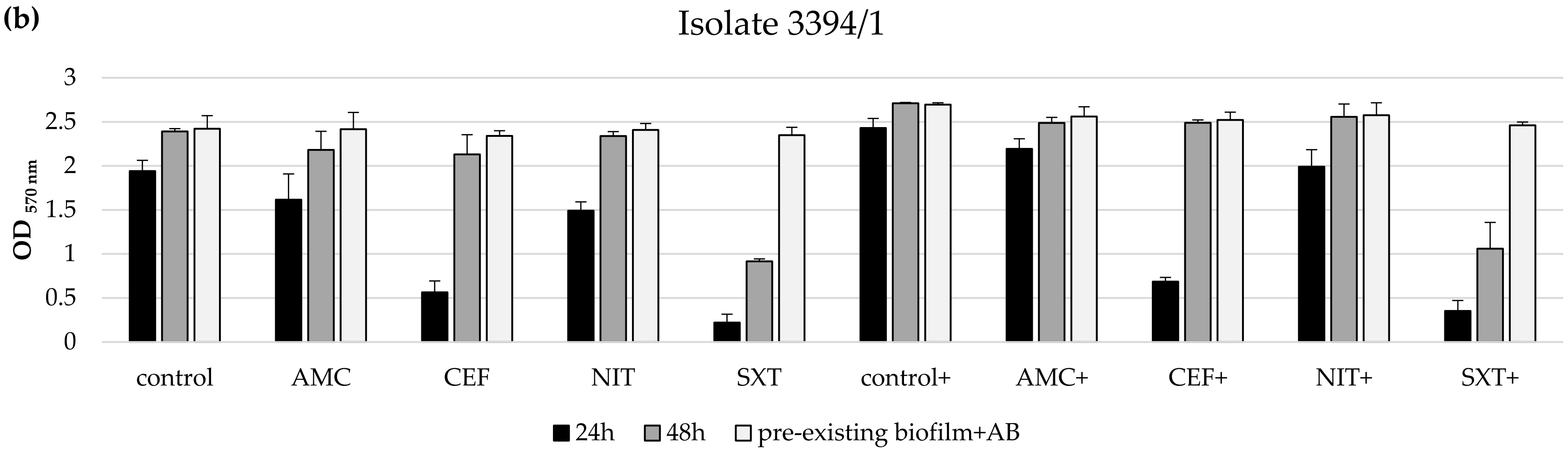

Isolate 3394/1
OD 570 nm
3
2.5
2
1.5
1
0.5
0
control
AMC
CEF
NIT
SXT
control+
AMC+
CEF+
NIT+
SXT+
24h
48h
pre-existing biofilm+AB

(c)

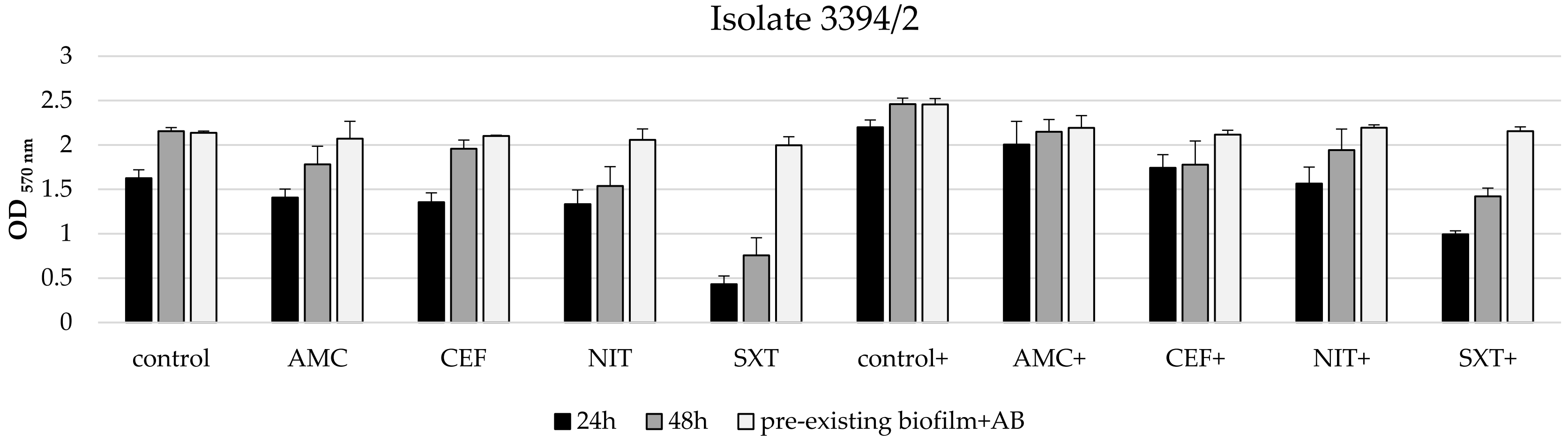
Isolate 3394/2
OD 570 nm
3
2.5
2
1.5
1
0.5
0
control
AMC
CEF
NIT
SXT
control+
AMC+
CEF+
NIT+
SXT+
24h
48h
pre-existing biofilm+AB

(d)

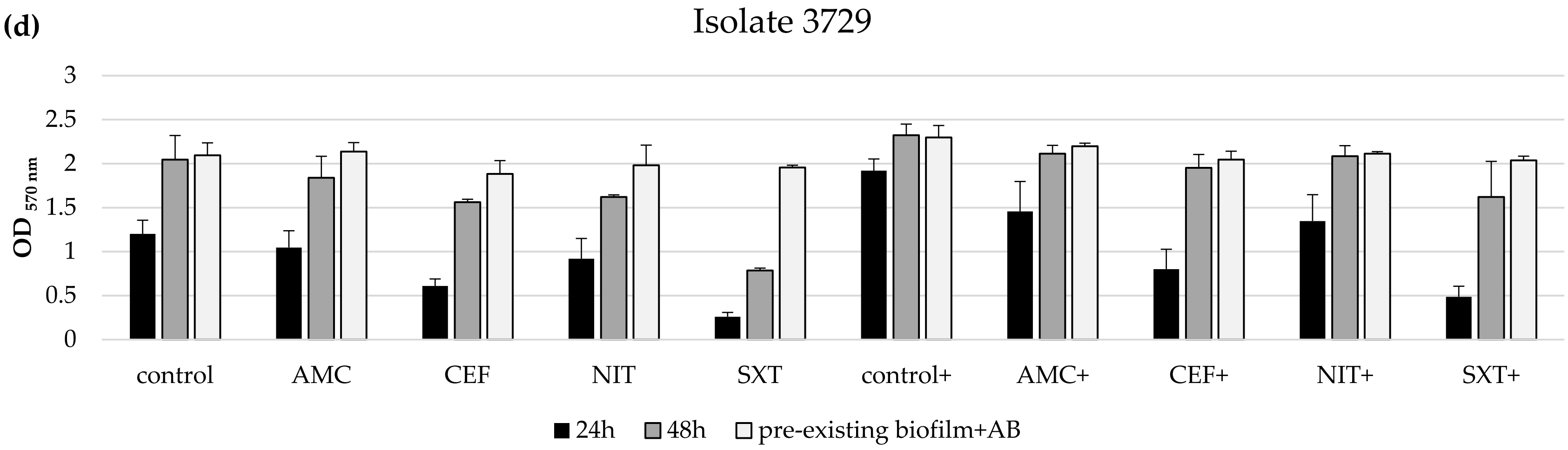
Isolate 3729
OD 570 nm
3
2.5
2
1.5
1
0.5
0
control
AMC
CEF
NIT
SXT
control+
AMC+
CEF+
NIT+
SXT+
24h
48h
pre-existing biofilm+AB

(e)

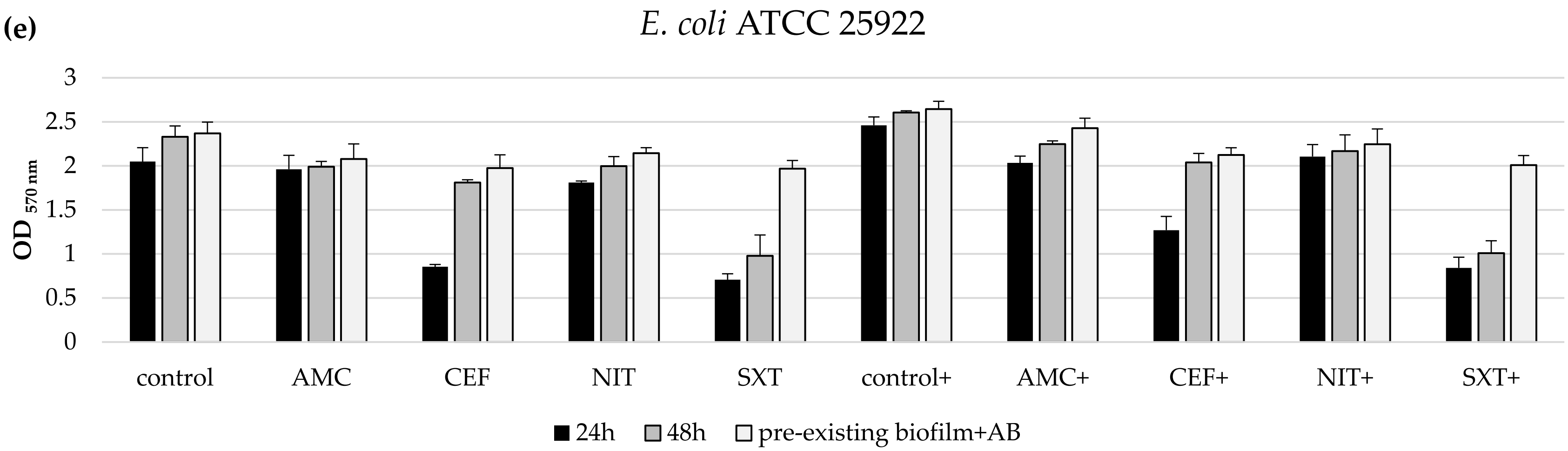

Figure S2. Effect of glucose (0 vs 1000 mg/dL) on biofilm biomass under different antibiotic exposure conditions in five *E. coli* isolates: (**a**) Isolate 2987; (**b**) Isolate 3394/1; (**c**) Isolate 3394/2; (**d**) Isolate 3729 and (**e**) *E. coli* ATCC 25922. Biofilms were treated with four antibiotics—amoxicillin-clavulanate (AMC), cefalexin (CEF), nitrofurantoin (NIT), and trimethoprim-sulfamethoxazole (SXT)—each at their MIC, in the absence (control) or presence of glucose (control+ and antibiotic+), under three experimental setups: (1) 24-h exposure, (2) 48-h exposure, and (3) antibiotic exposure after biofilm establishment (pre-existing biofilm + AB).
